# Supplementary material for: Neoptile feathers contribute to outline concealment of precocial chicks
Source: Sci Rep. 2021 Mar 9;11:5483. doi: 10.1038/s41598-021-84227-4 (PMC7943783; doi:10.1038/s41598-021-84227-4)
Supplement: Supplementary file 1 — Supplementary Information. [file 41598_2021_84227_MOESM1_ESM.pdf]

Neoptile feathers contribute to outline concealment of precocial chicks – Supplementary material

Authors: Veronika A. Rohr[1,2,*], Tamara Volkmer[3], Dirk Metzler[2], Clemens Küpper[1,*]

[1] Research Group for Behavioural Genetics and Evolutionary Ecology, Max Planck Institute for Ornithology, Seewiesen, Germany

[2] Division of Evolutionary Biology, Faculty of Biology, Ludwig-Maximilians-Universität München, Planegg-Martinsried, Germany

[3] Department of Migration, Max Planck Institute of Animal Behavior, Radolfzell, Germany

Mail: vrohr@orn.mpg.de; ckuepper@orn.mpg.de

Extended methods

Local Edge Intensity Analysis (LEIA)

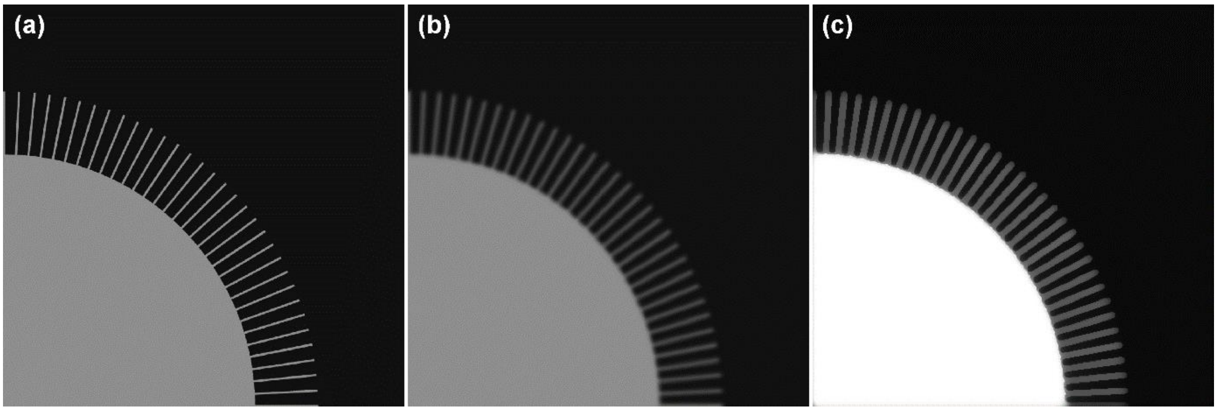

Figure S1: Modelling human vision with an acuity of 72 cpd. (a) Cone catch image (b) Gaussian Acuity Control (c) Edge reconstruction with Receptor Noise Limited (RNL) filter. All images exist as a stack of 4 channels (long, medium and short wave, and luminance). The figure was produced using Adobe Photoshop [1].

Table S1: Parameter combinations used in Experiment 1. Differences to Basic Scenario indicated in shaded cells

Parameter comb. index	Scenario	Number of appendages	Appendage thickness (Pt/px/mm)	Distance between appendages (px/mm)	Appendage transparency	Length hetero-geneity	Background	Acuity (cpd)
a		0		-				
b		32		88/7.5				
c		64		42/3.6				
d	Basic Scenario	128	1/4/0.4	19/1.6	0%	all 100% length	dark grey	72
e		256		7/0.6				
f		512		2/0.1				
g		full circle		-				
h		0		-				
i		32		84/7.1				
j		64		38/3.2				
k	Scenario 1a: 2 Pt Thickness	128	2/8/0.7	15/1.3	0%	all 100% length	dark grey	72
l		256		3/0.3				
m		512		-2/-0.2				
n		full circle		-				
o		0		-				
p		32		80/6.8				
q		64		34/2.9				
r	Scenario 1b: 3 Pt Thickness	128	3/12/1.1	11/0.9	0%	all 100% length	dark grey	72
s		256		-1/-0.1				
t		512		-7/-0.6				
u		full circle		-				
v		0		-				
w		32		88/7.5				
x		64		42/3.6				
y	Scenario 2a: 25% Transparency	128	1/4/0.4	19/1.6	25%	all 100% length	dark grey	72
z		256		7/0.6				
aa		512		2/0.1				
ab		full circle		-				
ac		0		-				
ad		32		88/7.5				
ae		64		42/3.6				
af	Scenario 2b: 50% Transparency	128	1/4/0.4	19/1.6	50%	all 100% length	dark grey	72
ag		256		7/0.6				
ah		512		2/0.1				
ai		full circle		-				

aj		0		-				
ak		32		88/7.5				
al		64		42/3.6				
am	Scenario 3a: 1/2 at 50% length	128	1/4/0.4	19/1.6	0%	1/2 at 50% length	dark grey	72
an		256		7/0.6				
ao		512		2/0.1				
ap		full circle		-				
aq		0		-				
ar		32		88/7.5				
as		64		42/3.6				
at	Scenario 3b: 1/2 at 25% & 1/4 at 50% length	128	1/4/0.4	19/1.6	0%	1/2 at 25% & 1/4 at 50% length	dark grey	72
au		256		7/0.6				
av		512		2/0.1				
aw		full circle		-				
ax		0		-				
ay		32		88/7.5				
az		64		42/3.6				
ba	Scenario 4a: background - big tiles	128	1/4/0.4	19/1.6	0%	all 100% length	chessboard fields 346 px/29.3 mm	72
bb		256		7/0.6				
bc		512		2/0.1				
bd		full circle		-				
be		0		-				
bf		32		88/7.5				
bg		64		42/3.6				
bh	Scenario 4b: background - small tiles	128	1/4/0.4	19/1.6	0%	all 100% length	chessboard fields 86 px/7.3 mm	72
bi		256		7/0.6				
bj		512		2/0.1				
bk		full circle		-				
bl		0		-				
bm		32		88/7.5				
bn		64		42/3.6				
bo	Scenario 5a: 30 cpd acuity	128	1/4/0.4	19/1.6	0%	all 100% length	dark grey	30
bp		256		7/0.6				
bq		512		2/0.1				
br		full circle		-				
bs		0		-				
bt		32		88/7.5				
bu		64		42/3.6				
bv	Scenario 5b: 10 cpd acuity	128	1/4/0.4	19/1.6	0%	all 100% length	dark grey	10
bw		256		7/0.6				
bx		512		2/0.1				
by		full circle		-				

Table S2: Spatial acuity of humans and potential chick predators.

Spatial acuity approximation	Potential predator of snowy plover chicks	Related species with known spatial acuity	Spatial acuity
High (72 cpd)	human *(Homo sapiens)*	human (*Homo sapiens*) – [2–5]	72 – 73 cpd
	birds of prey [6,7]	brown falcon (*Falco berigora*) – [8]	73 cpd
Medium (30 cpd)	crested caracaras (*Caracara cheriway*) – (C Küpper, personal observations)	chimango caracara (*Phalcoboenus chimango*) – [9,10]	15 – 40 cpd
	corvids – [6,7]	several corvids – [10,11]	30 – 33 cpd
	racoon (*Procyon lotor*) – [12]	racoon (*Procyon lotor*) – [13]	25 – 30 cpd
Low (10 cpd)	feral dog (*Canis familiaris*) – [12]	dog (*Canis familiaris*) – [14–16]	4.62 – 12.59 cpd
	coyote (*Canis latrans*) – [12]		
	bobcat (*Lynx rufus*) – [12]	European lynx (*Lynx europea*) – [17]	7 – 8 cpd
		cat (*Felis catus*) – [2,18]	10 cpd

Mean Luminance Comparison (MLC)

Experiment 1: Proof of principle

For the Mean Luminance Comparison (MLC), we analysed the same images as with the LEIA. We divided the filtered image into three regions of interest (ROIs) (Fig. S3a). 1) The object region included the whole object inside up to 20 pixels next to the object outline. 2) The appendage region was an 80 pixel-wide band including only the area covered by appendages. It started 20 pixels outside the object outline and reached up to 20 pixels before the boundary created by the appendages (appendage-boundary). 3) The background region ranged from 20 pixels outside the appendage-boundary to a 1500x1500 pixel-wide rectangle surrounding the object. A buffer zone of 40 pixels between all three regions was excluded from the analysis to ensure a clear separation of the regions. In the luminance channel of each image, we measured the mean luminance in the three regions and compared them subsequently. Luminance values range from 0 to 1.

According to background matching, objects that differ more in luminance from the background are more conspicuous and hence less well camouflaged [19]. We assumed that detectability based on possible luminance differences between object and background are weakened by the appendages as they form a transition zone helping to blend the object better into the background. Accordingly, from

a camouflage perspective, the appendage region would provide an optimal transition zone when its mean luminance is exactly the mean of the object and background region's luminance.

Experiment 2: Chick photographs

Similar to the artificial object experiment, the chick region included everything inside the chick outline, the background region included everything outside the feather-boundary up to a 1500x1500 pixel-wide rectangle surrounding the chick and the feather region (FR) was between chick outline and feather-boundary. Note that the FR is different from the contour region, which additionally includes a small part of chick and background region. We reduced the FR by excluding all areas that were shaded by the chick since this shadow was missing on the empty background images. Additionally, we excluded the buffer zone (Fig. S3a, the area between the coloured regions) to cover the whole variation in feather density in the FR (Fig. S4a-b). Close to the chick outline, the feathers were still relatively dense thinning more and more towards the feather-boundary as they were very variable in length.

For each chick, we measured the mean luminance of all three regions in the luminance channel of the image containing the chick without feathers (Fig. S4a). The FR we measured again in the image containing the chick with feathers (Fig. S4b).

In theory, the best transition zone between chick and background, i.e., the zone that reduces the outline of the chick against the background the most, should have an exactly intermediate luminance between chick and background region. In a first analysis, we checked whether the absolute distance of mean luminance of the FR with feathers was closer to those optimal values than without feathers. Because the luminance data were not normally distributed according to the Shapiro-Wilk normality test we conducted a Wilcoxon paired signed rank test. To compare the data graphically in an intuitive way, we transformed the values so that the chick region always was the reference with a value of 0, the background region became 1. The two values measured in the FR stayed in their initial relative distance to chick and background value.

The FR generally was quite narrow compared to chick and background region and its effect probably acts predominantly from close proximity. Therefore, we focussed the next analysis only on chick and FR. We assumed that the chick to a certain extent differs in luminance from its immediate background in the FR and that including the feathers decreases this difference and thus possibly improves the camouflage. Therefore, we compared the absolute distances between the mean luminance of chick region and FR with and without feathers. As the data were normally distributed according to the Shapiro-Wilk normality test we conducted a two-sided paired t-test.

For an easier comparison of the measurements, we transformed the luminance values in this analysis. The chick region again was the reference with a value of 0. As the background region was excluded,

we scaled the FR without feathers to 1. The value measured in the FR with feathers stayed in its initial relative distance to the other two values.

The analysis aimed to check if the FR meets the basic requirement of a transition zone having intermediate luminance. Thus, we checked whether the mean luminance value of the FR with feathers fell between the one of chick region (mean luminance = 0) and FR without feathers (mean luminance = 1) constituting the immediate surrounding background to account for the local scale. We calculated the probability for the FR with feathers of having a value between 0 and 1 when randomly distributed. For this, we drew a random sample (n = 10,000) from a normal distribution with the mean and standard deviation in the transformed data. Then, we ran an exact binomial test to determine whether the observed intermediate luminance value was different from the expected value.

Extended results

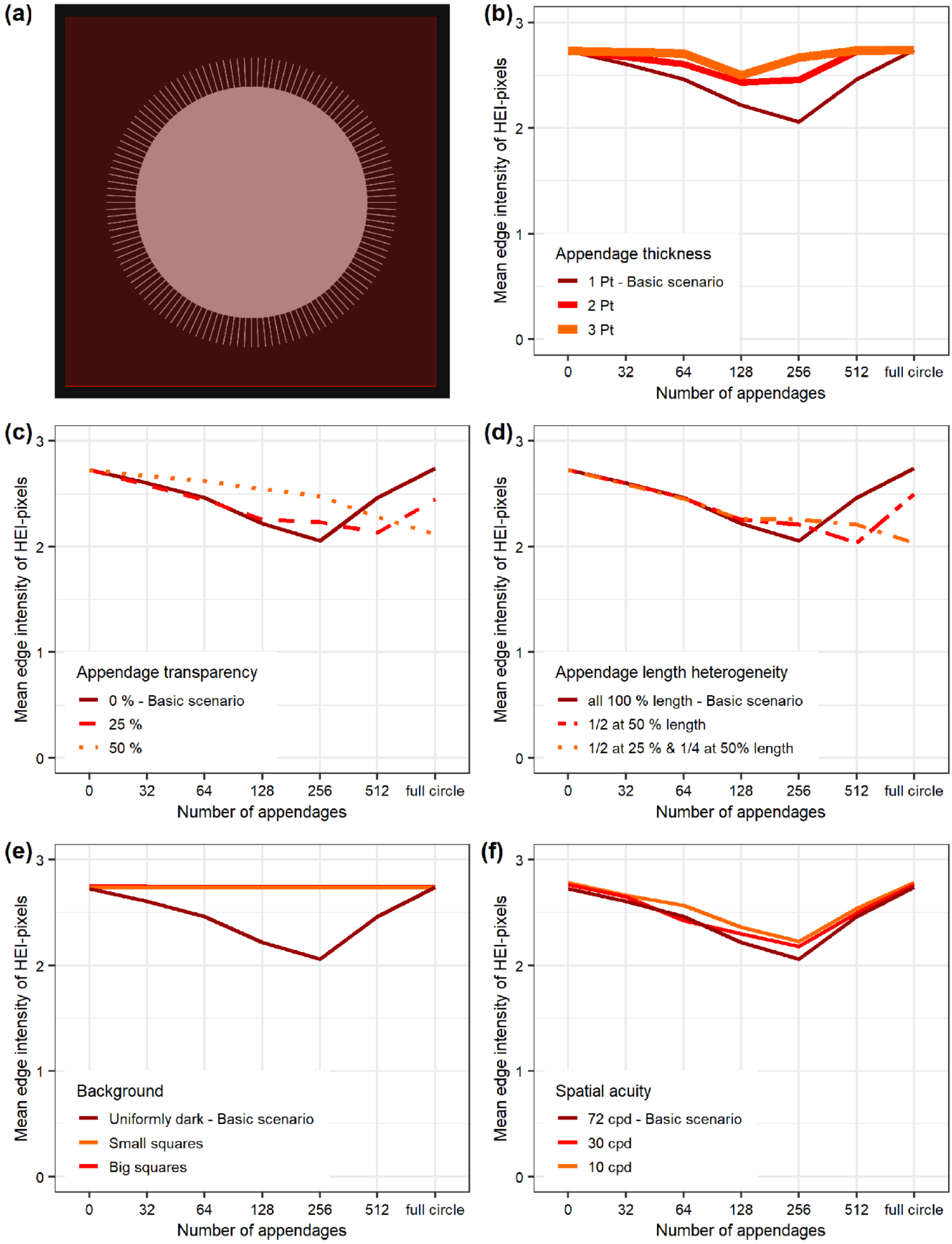

Figure S2: Local edge intensity analysis (LEIA) in experiment 1 with an expanded region of interest (ROI). The highest 0.37 % of the pixels are classified as “High Edge Intensity-pixels” (HEI-pixels). (a) ROI is a 1500 x 1500 pixel-wide rectangle (red). (b) Scenario 1: the Basic Scenario (darkest red) altered towards thicker appendages. (c) Scenario 2: the Basic Scenario altered towards more transparent appendages. (d) Scenario 3: the Basic Scenario altered towards a more heterogeneous appendage length. (e) Scenario 4: the Basic Scenario altered

towards higher background complexity. (f) Scenario 5: the Basic Scenario altered towards lower spatial acuity. The figure was produced using R [20] and Adobe InDesign [21].

Table S3: General characterisation of the chick images. Percentage values of ROI available for analysis.

Chick ID	Feather region excl. shadow	Contour region excl. shadow	HEI-pixels threshold
CN0333	77%	75%	0.9859
CN0339	57%	58%	0.9827
CN0340	61%	62%	0.9789
CN0345	88%	82%	0.9815
CN0347	61%	59%	0.9839
CN0350	82%	82%	0.9829
CN0353	91%	89%	0.9789
CN0356	58%	58%	0.9830
CN0360	64%	64%	0.9854
CN0361	100%	100%	0.9840
CN0363	100%	100%	0.9789
CN0364	81%	78%	0.9846
CN0367	58%	56%	0.9858
CN0411	66%	56%	0.9821
CN0415	57%	55%	0.9800
Mean	73%	72%	0.9826

For eight of the 15 analysed chicks, the empty background image was slightly shifted because of a camera movement. Therefore, we corrected their position manually to place the chicks exactly at the same spot in the empty background.

Mean Luminance Comparison (MLC)

Experiment 1: Proof of principle

The mean luminance of the area covered by appendages (appendage region) was generally intermediate between the luminance of object and background across all scenarios indicating the formation of a luminance transition zone (Fig. S3b-f). Without appendages, the appendage region's mean luminance was the same as the one of the background region. With an increasing number of appendages, the appendage region's mean luminance became more and more similar to the object region's luminance until they were identical when the appendages formed a full circle (Fig. S3b, dark green curve).

Appendage characteristics

Increasing the appendage thickness in Scenario 1 caused the appendage region's luminance to converge sooner with the object region's luminance. The optimum was also reached sooner, between 128 and 256 appendages at 2 Pt thickness and around 128 appendages at 3 Pt thickness respectively

(Fig. S3b). Having 128 appendages of 3 Pt thickness was the best parameter combination tested. In this image, approximately 50 % of the appendage region's area was covered with appendages. This suggests for the basic scenario that the optimal intermediate luminance would have been reached for objects that have between 256 and 512 appendages (Fig. S3b, '1Pt'), when 50 % of the appendage region would have been covered by appendages. In contrast, with increasing appendage transparency (Scenario 2) more appendages were needed to reach the same luminance values compared to the Basic Scenario. At 25 % transparency, the full circle of appendages was needed to reach the optimum intermediate value and with 50% transparency, the intermediate value could not be reached at all (Fig. S3c). Similarly, with increasing appendage length heterogeneity (Scenario 3) more appendages were required to reach the optimum but it was obtained when half of the appendages had 50 % of the length as well as when half of the appendages had 25 % and a quarter had 50 % of the length (S3d).

Background complexity and spatial acuity

Increasing the background complexity did not affect the curve trajectories in the transition zone. Without appendages, the appendage region's mean luminance was similar to the background's luminance and became increasingly similar to the object's luminance when raising the number of appendages until they converged with a full circle of appendages (Fig. S3e). Likewise, lowering the spatial acuity in Scenario 4 did not clearly change the curve trajectory in the transition zone (Fig. S3f).

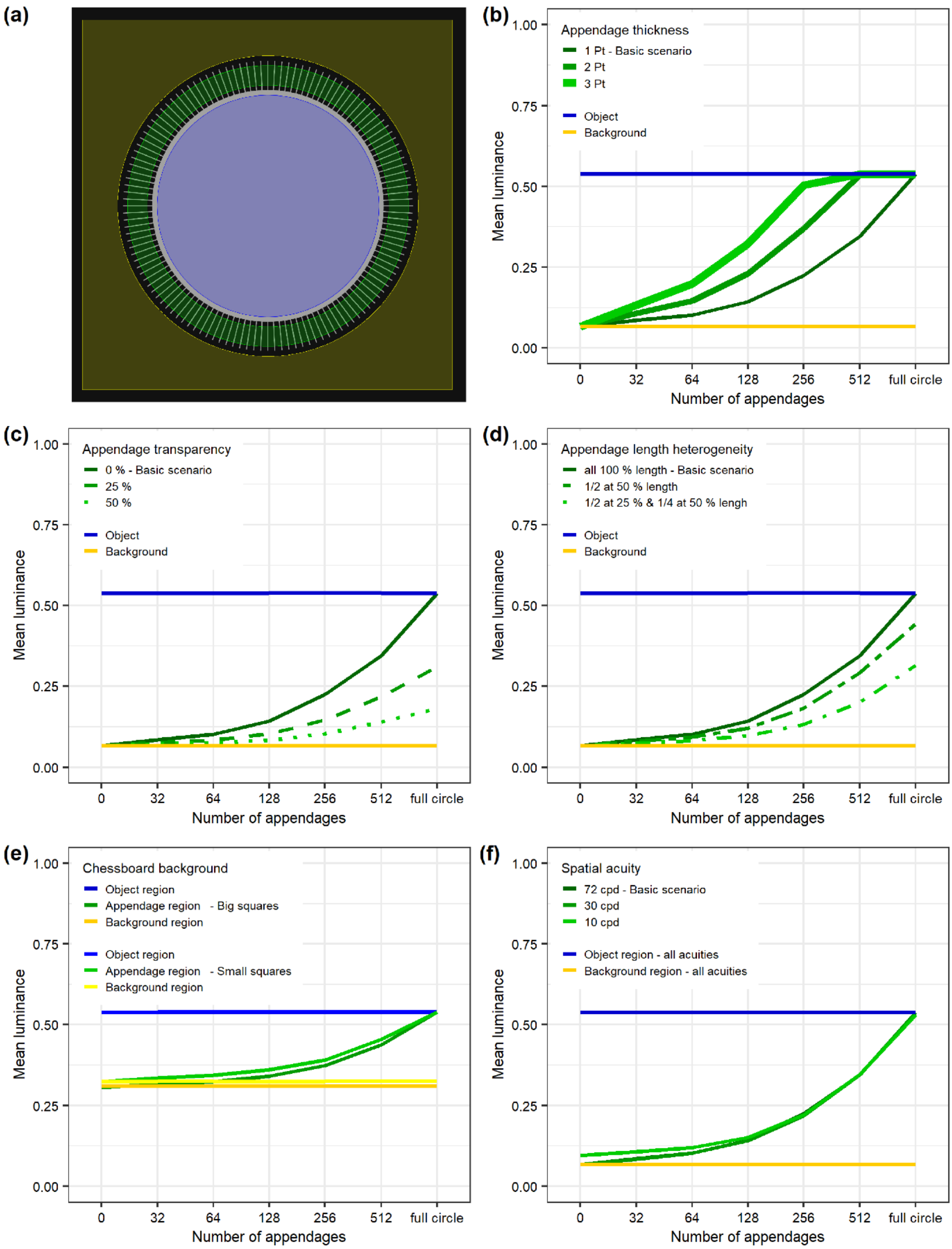

Figure S3: Mean Luminance Comparison in experiment 1. (a) The three regions of interest (ROIs) analysed were object region (blue), background region (yellow) and appendage region (green). The ROIs were separated by 40 pixels to ensure a clear separation of the regions. (b) Scenario 1: variation in appendage thickness. (c) Scenario 2: variation in appendage transparency. (d) Scenario 3: variation in appendage length. (e) Scenario 4: variation in background complexity. (f) Scenario 5: variation in spatial acuity. Note that the 30 and 72 cpd curves overlap fully. The figure was produced using R [20] and Adobe InDesign [21].

Experiment 2: Chick photographs

For the MLC, presence of protruding neoptile feathers did not contribute to creating a transition zone between chick and background, as we did not observe more intermediate mean luminance values in the ROI in comparison to ROI of chick pictures without protruding feathers (Fig. S4c-f).

After removing the areas that were shaded by the chicks, on average 73 % of the FR remained for the MLC (Table S3). This value was slightly different from the 72 % that remained of the contour region in the LEIA because FR and contour region differed in size and the extent to which they were shaded by the chick.

Presence of feathers did not change the mean luminance of the transition zone adaptively. The distance of the mean luminance of the FR to the optimal intermediate value was in 9 of 15 chicks (60 %) shorter with than without feathers (Fig. S4c-d). The Wilcoxon paired signed rank test showed no clear difference between the distribution of the two groups ($p = 0.45$).

Feathers did not make mean luminance of the FR more similar to the mean luminance of the chick region. Although the distance of the mean luminance of the FR with feathers to the mean luminance of the chick region was in 10 of 15 cases (66.7 %) shorter than without feathers (Fig. S4f). There was no clear difference between images with and without protruding feathers ($t = 1.1263$, $df = 14$, $p = 0.28$). The mean luminance difference between the measurements with and without feathers was 0.0077 (95 %CI: -0.0070, 0.0224).

The FR with feathers had an intermediate mean luminance between the chick region and the FR without feathers in 8 of 15 chicks (53 %) (Fig. S4e). In the random sample ($n = 10,000$) from a normal distribution with the same mean and standard deviation as observed in the transformed data, 38 % of the values were intermediate between 0 and 1. Including the protruding feathers, we observed a proportion of 0.53 (95 %CI: 0.27, 0.79) intermediate values, however, this was not clearly different from expected by chance ($p = 0.29$).

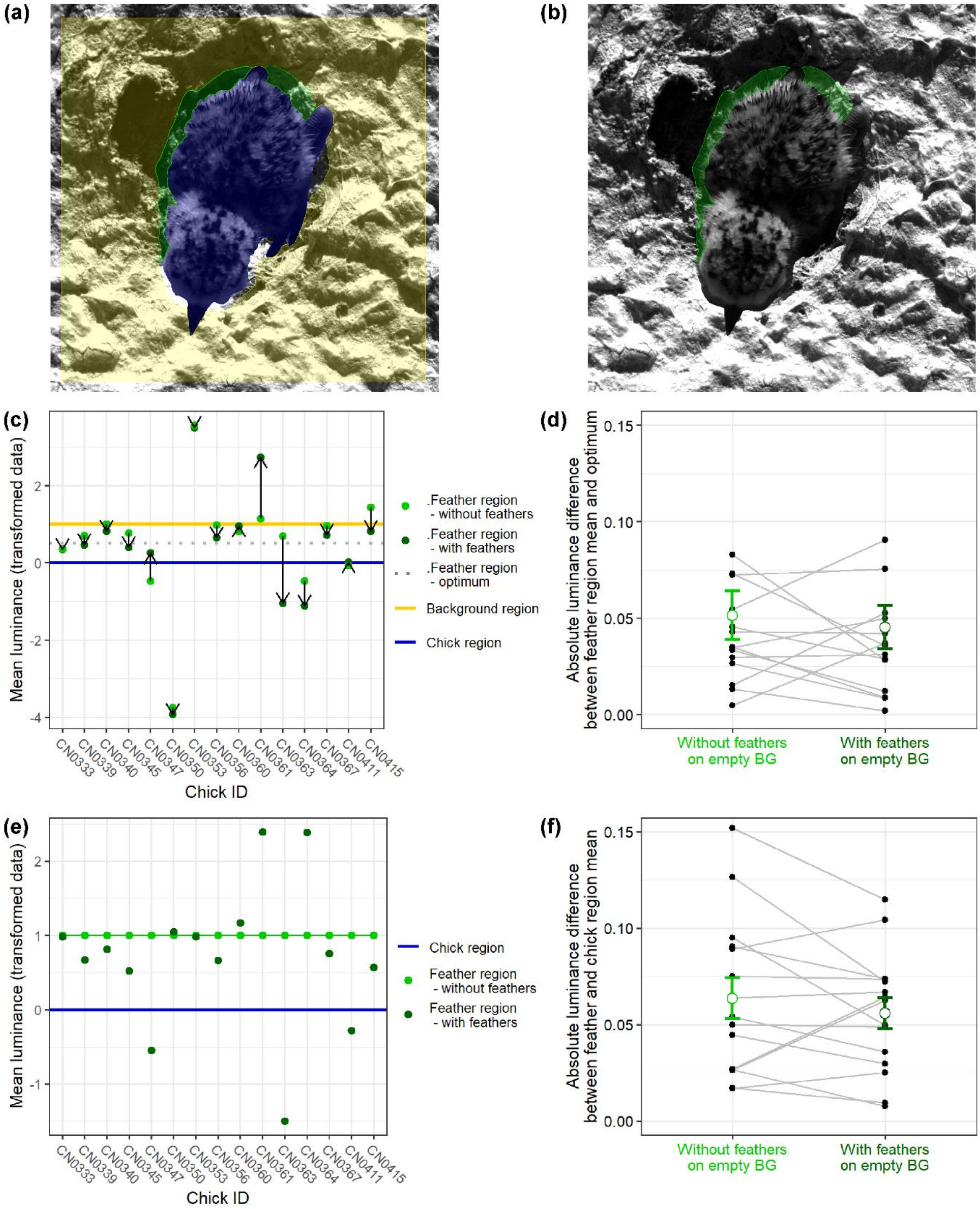

Figure S4: Mean Luminance Comparison in experiment 2. The regions of interest (ROIs) analysed were (a) chick (blue) background (yellow) and feather region (FR) without feathers (green) measured in scene 2 and (b) the FR with feathers (green) measured in scene 1. (c) Measurements were transformed so that the chick region (blue) was always “0” and the background region (yellow) “1”. The FR optimum (grey, dotted) portrays the mean of chick and background region. The arrows indicate the direction in which the value of the FR was shifted when the feathers were present. (d) Absolute luminance difference between FR mean and optimum with and without feathers.* (e) Measurements were transformed so that the chick region (blue) was always “0” and the FR without feathers (light green) “1”. (f) Absolute luminance difference between chick region and FR mean with and without feathers.* The figure was produced using R [20] and Adobe InDesign [21].

*Measurements are paired by chick ID. The error bars indicate group mean +/- standard error.

References

References

1. Adobe Inc. Adobe Photoshop CS6. Version 13.0 x64. https://www.adobe.com/products/photoshop.html (2012).
2. Caves, E. M. & Johnsen, S. AcuityView: an r package for portraying the effects of visual acuity on scenes observed by an animal. *Methods Ecol. Evol.* **9,** 793–797 (2018).
3. Land, M. F. Optics and vision in invertebrates. *Handb. Sens. Physiol.* **VII/6B,** 471–592 (1981).
4. Land, M. F. & Nilsson, D.-E. *Animal eyes* (Oxford Univ. Press., 2012).
5. Hirsch, J. & Curcio, C. A. The spatial resolution capacity of human foveal retina. *Vision Res.* **29,** 1095–1101 (1989).
6. Page, G. W., Stenzel, L. E. & Ribic, C. A. Nest site selection and clutch predation in the snowy plover. *Auk* **102,** 347–353 (1985).
7. Mabee, T. J. & Estelle, V. B. Assessing the effectiveness of predator exclosures for plovers. *Wilson Bull.* **112,** 14–20 (2000).
8. Reymond, L. Spatial visual acuity of the falcon, *Falco berigora*: A behavioural, optical and anatomical investigation. *Vision Res.* **27,** 1859–1874 (1987).
9. Potier, S., Bonadonna, F., Kelber, A. & Duriez, O. Visual acuity in an opportunistic raptor, the chimango caracara (*Milvago chimango*). *Physiol. Behav.* **157,** 125–128 (2016).
10. Caves, E. M., Brandley, N. C. & Johnsen, S. Visual acuity and the evolution of signals. *Trends Ecol. Evol.* **33,** 358–372 (2018).
11. Dabrowska, B. Investigations on visual acuity of some corvine species. *Folia Biol.* **23,** 311 (1975).
12. Stoddard, M. C. *et al.* Camouflage and clutch survival in plovers and terns. *Sci. Rep.* **6,** 32059 (2016).
13. Johnson, J. I. & Michels, K. M. Discrimination of small intervals and objects by raccoons. *Anim. Behav.* **6,** 164–170 (1958).
14. Bromberg, N. M. & Dawson, W. W. Preliminary measures of canine visual spatial resolution with electrophysiological techniques. *Trans. Am. Coll. Vet. Ophth. Soc.* **11,** 120–125 (1980).
15. Odom, J. V., Bromberg, N. M. & Dawson, W. W. Canine visual acuity: retinal and cortical field potentials evoked by pattern stimulation. *Am. J. Physiol.* **245,** R637-41 (1983).
16. Pretterer, G., Bubna-Littitz, H., Windischbauer, G., Gabler, C. & Griebel, U. Brightness discrimination in the dog. *J. Vis.* **4,** 241–249 (2004).
17. Maffei, L., Fiorentini, A. & Bisti, S. The visual acuity of the lynx. *Vision Res.* **30,** 527–528 (1990).
18. Wässle, H. Optical quality of the cat eye. *Vision Res.* **11,** 995-IN14 (1971).

19. Endler, J. A. An overview of the relationships between mimicry and crypsis. *Biol. J. Linn. Soc. Lond.* **16,** 25–31 (1981).
20. R Core Team. R. A language and environment for statistical computing. https://www.R-project.org (R Foundation for Statistical Computing, 2019).
21. Adobe Inc. Adobe InDesign CS6. Version 8.0. https://adobe.com/products/indesign.html (2012).
